# Utilization of Portable Brain Magnetic Resonance Imaging in an Acute Care Setting

**DOI:** 10.7759/cureus.33067

**Published:** 2022-12-28

**Authors:** Alice Wang, Imran Siddiqi, Maxwell A Marino, Lana Maniakhina, Jeffrey J Li, Andrew Ku, Katherine Ko, Dan E Miulli

**Affiliations:** 1 Department of Neurological Surgery, Riverside University Health System Medical Center, Moreno Valley, USA; 2 Department of Internal Medicine, Edward Via College of Osteopathic Medicine, Spartanburg, USA; 3 Department of Internal Medicine, Cleveland Clinic Indian River Medical Center, Vero Beach, USA; 4 Department of Internal Medicine, California University of Science and Medicine, Colton, USA; 5 Department of Neurosurgery, California University of Science and Medicine, Colton, USA; 6 Department of Neurosurgery, Arrowhead Regional Medical Center, Colton, USA

**Keywords:** neurological surgery, neurosurgery, portable mri, mri, magnetic resonance imaging

## Abstract

Background

Magnetic resonance imaging (MRI) is an important noninvasive diagnostic tool used in multiple facets of medicine, especially in the assessment of the neurological system with increasing usage over the past decades. Advancement in technology has led to the creation of portable MRI (pMRI) that was cleared for use recently.

Methodology

A prospectively collected retrospective study was conducted at a single institution to include patients aged >18 years, admitted to the hospital, and requiring MRI for any brain pathology. pMRI was completed using portable MRI. Traditional MRI was completed with a standard 1.5T MRI, and when possible, the results of the two studies were compared.

Results

We obtained pMRI on 20 patients, with a total of 22 scans completed. Notably, on the pMRI, we were able to identify midline structures to determine midline shifts, identify the size of ventricles, and see large pathologies, including ischemic and hemorrhagic strokes, edema, and tumors. Patients with implants or electrodes in and around the calvarium sometimes pose challenges to image acquisition.

Conclusions

Portable brain MRI is a practical and useful technology that can provide immediate information about the head, especially in an acute care setting. Portable brain MRI has a lower resolution and quality of imaging compared to that of transitional MRI, and therefore, it is not a replacement for traditional MRI.

## Introduction

Magnetic resonance imaging (MRI) is an important noninvasive diagnostic tool used in multiple facets of medicine, especially common in neurological surgery [1-3]. Its use has increased by nearly threefold from 2003 to 2013 and has been increasing since throughout hospital systems [4]. The clarity of neuronal structures on MRIs has contributed to their increased use during intraoperative neurosurgeries [5,6] and even in the acute emergency setting for their advantages in soft tissue contrast. However, many facilities have limited access to using MRIs because of their high costs and low portability [7,8]. To address this, the advancement in technology has led to the creation of portable MRI (pMRI) that was cleared for use within the past couple of years. The utilities of this new and advanced device are still being investigated. One study found pMRI to be effective in assessing brain injuries in critically ill patients diagnosed with COVID-19 [9]. Other studies have remarked on the safety, feasibility, and ease of obtaining point-of-care MRIs in various clinical settings using pMRI [10-14]. Furthermore, traditional MRI machines have a magnetic field of 1.5 or 3 T [15-16]. pMRI has a low magnetic field between 50 and 200 mT [17-18]. This enables small, metallic objects to be used in conjunction with pMRI without risk, as was shown in evidence summaries when the electrocardiogram (ECG) leads were kept attached in over 210 patients [19-24].

We explored pMRI as well as an array of devices for their concomitant use in the neurological surgery patient. Assessment devices such as an electroencephalogram (EEG), Licox, or halo brace within pMRI are important because these devices are known to limit standard MRI imaging. pMRI can be brought to the bedside, and patients can be scanned even when unstable for transport. Without the need to transport the patient, we hypothesize that there will be a decrease in the burden on limited resources, which includes freeing up MRI availability, awaiting respiratory therapy (RT) support, and more. At the same time, this may increase the number of patients who can receive MRI due to the decreased patient-to-scan time. This study sought to determine the feasibility of using pMRI by evaluating the interpretation of such scans and, where possible, comparing them to traditional MRI. 

## Materials and methods

This study was approved by the Arrowhead Regional Medical Center (ARMC) Institutional Review Board (IRB), protocol #22-45. The study design was that of a prospectively collected retrospective study throughout 2022 at ARMC. Imaging studies were evaluated by resident neurosurgery physicians and verified by attending neurosurgery physicians. Inclusion criteria consisted of patients aged >18 years, admitted to the hospital, and requiring MRI for any brain pathology. Patients without the need for MRI were excluded. pMRI was completed using portable MRI (Swoop™ Hyperfine, Guilford, CT, USA). Traditional MRI was completed with a standard 1.5 T MRI. When appropriate, patients had both pMRI and traditional MRI completed as close together as possible. If any scan was unable to be fully completed between pMRI and traditional MRI, this data was not used. 

## Results

**Figure 1:**
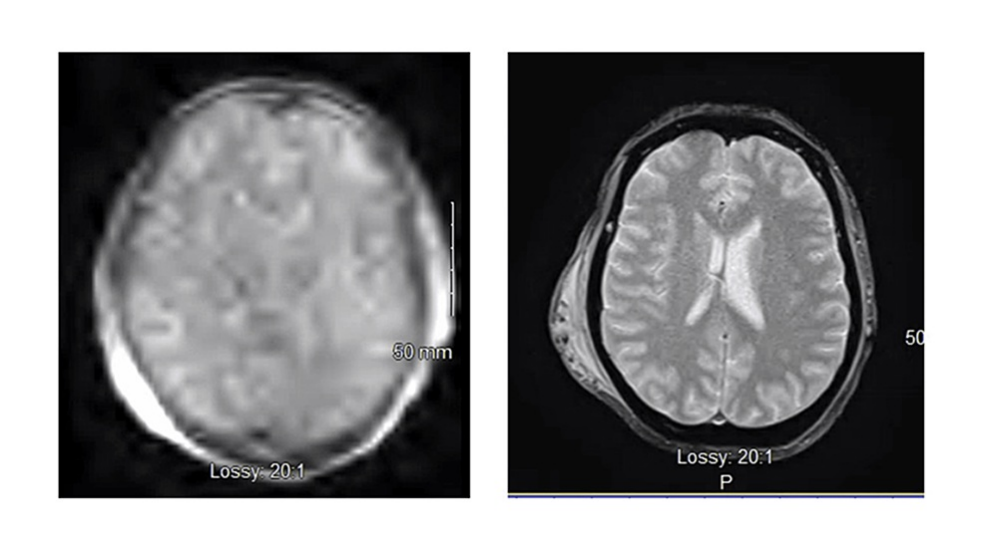
T2 axial MRI. Left - pMRI; right - traditional T2 MRI of the same patient, status post-trauma with associated extracalvarial hematoma. MRI, magnetic resonance imaging; pMRI, portable magnetic resonance imaging

**Figure 2:**
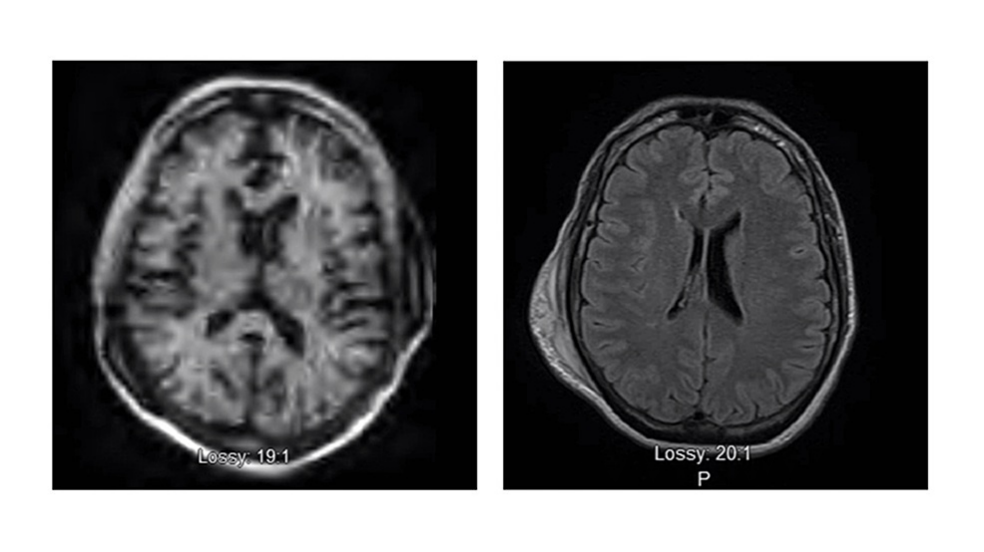
T1 axial MRI. Left - pMRI; right - traditional T1 MRI of the same patient. MRI, magnetic resonance imaging; pMRI, portable magnetic resonance imaging

We obtained pMRI on 20 patients, totaling 22 scans. Many of these patients on the neurosurgery service were unable to undergo traditional MRI, so a decision to obtain pMRI was made. Notably, on the pMRI, we were able to identify midline structures to determine midline shifts, identify the size of ventricles, and see large pathologies, including ischemic and hemorrhagic strokes, edema, and tumors. A representative patient with both pMRI and traditional MRI, respectively, is seen in Figures 1 and 2.

**Figure 3:**
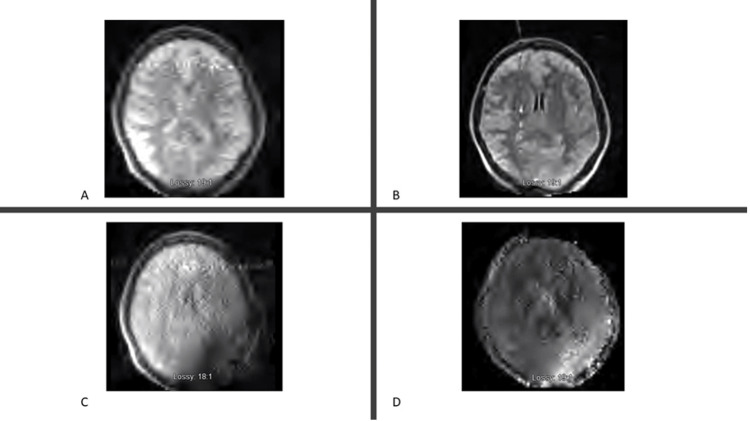
pMRI of the patient with an EEG lead placement. (A) Axial T2. (B) Axial T1. (C) Axial T1 FLAIR. (D) DWI. EEG, electroencephalography; pMRI, portable magnetic resonance imaging; FLAIR, fluid level attenuated inversion recovery; DWI, diffusion-weighted imaging

Patients with implants or electrodes in and around the calvarium sometimes pose challenges to image acquisition. In this study, one patient with electroencephalography (EEG) lead placement underwent pMRI, as shown in Figure 3.

 

**Figure 4:**
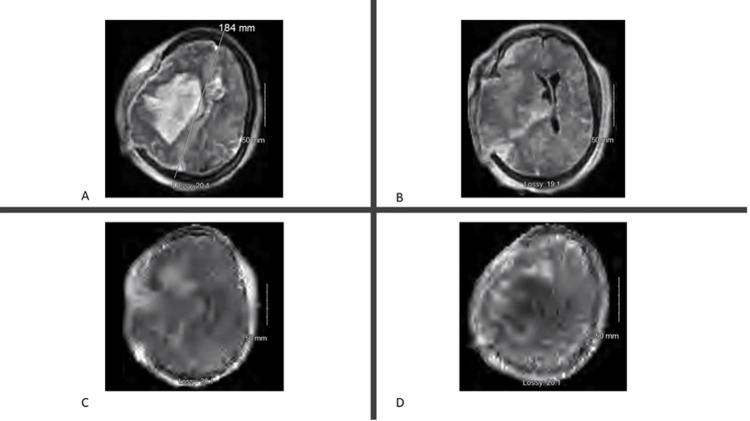
pMRI of the patient with a hemorrhagic stroke. (A) Axial T2. (B) Axial T1. (C) Axial T1 FLAIR. (D) DWI. pMRI, portable magnetic resonance imaging; FLAIR, fluid level attenuated inversion recovery; DWI, diffusion-weighted imaging

Additionally, one patient with hemorrhagic stroke who underwent craniectomy had a pMRI shown in Figure 4.

We found that we were able to safely use pMRI concurrently with EEG without disrupting the integrity of the images. However, Licox and the halo brace were incompatible with pMRI because patients with these devices were unable to fit into the pMRI brain coil.

## Discussion

Traditional MRI machines have a magnetic field of 1.5 or 3 T [15,16]. pMRI has a lower magnetic field between 50 and 200 mT [17,18]. The decreased strength of the magnet in pMRI translates to lower resolution and lower quality images compared to traditional MRI. The tradeoff in the quality of the image and ability to perform the study with small metallic devices to the ease of obtaining the scan needs to be weighed against the improved resolution of traditional MRI. Quality of imaging may not be the priority in certain clinical settings where the urgency of the scan, stability of the patient, or type of pathology is of foremost importance. A potential approach to this problem would be implementing super-resolution techniques, which may be available soon with the current technological advancements [19].

There are several advantages to the utilization of pMRI. First, the pMRI machine produces images that accurately, though not as precisely, reflect the conditions within the calvarium. Other studies have also shown that the pMRI machine produced useful images of the brain [17]. In this paper, we showed that pMRI is useful in situations where patients are hemodynamically unstable and therefore unable to undergo traditional MRI (the case in only some of our patients). pMRI was obtained in patients with EEG leads on their scalps. In contrast, traditional MRI requires the removal of the leads. Another advantage of pMRI is having the patient remain in the same bed without the need to transfer, thus enabling imaging of patients with higher weights who are deemed unsuitable for traditional MRI. The wait for a transport team to bring the patient to the MRI suite is also decreased, which is especially critical in time-sensitive settings. In addition, training is not required to move pMRI, so any hospital staff can bring the pMRI machine to the bedside. Finally, pMRI, unlike CT, does not increase radiation exposure in patients.

The utilization of pMRI in other clinical settings warrants further investigations. Most hospitals do not have a pMRI machine. The few hospitals with pMRI, including Riverside Community Hospital, may not have established protocols for when to use it. Protocols at our institution are currently being finalized to streamline the use and availability of pMRI.

There are some limitations of this study. This is a single-center institution study, and the sample size is small. The latter may be due to a lack of protocol stating the clinical scenarios to use pMRI at our institution. The pMRI machine also requires a trained operator who may not be available at the time that pMRI is needed. Some providers are unfamiliar with pMRI, and therefore, they avoid ordering pMRI in clinical scenarios where pMRI would have been helpful in patient care. Future studies should address the aforementioned limitations, with a focus on recruitment of other institutions, the establishment of pMRI protocols, training and hiring of more operators, and increasing familiarity with pMRI. Future directions of study should seek to get the measurements for midline shifts between pMRI and traditional MRI with associated kappa statistics for different observers.

## Conclusions

The portable brain MRI is a practical and useful technology to provide immediate information about the head, especially in an acute care setting. Portable brain MRI has a lower resolution and quality of imaging compared to transitional MRI, and therefore, it is not a replacement for traditional MRI. pMRI can be helpful in time-sensitive situations when patients are unable to withstand traditional MRI. This study supports the use of pMRI in patients with EEG leads but not with Licox or halo brace. More studies are needed to investigate the compatibility of pMRI in other clinical settings, as well as a direct comparison of metrics between the two studies.
